# Supplementary figures and images for: Comparative Transcriptomics Sheds Light on Remodeling of Gene Expression during Diazotrophy in the Thermophilic Methanogen Methanothermococcus thermolithotrophicus
Source: mBio. 2022 Nov 21;13(6):e02443-22. doi: 10.1128/mbio.02443-22 (PMC9765008; doi:10.1128/mbio.02443-22)

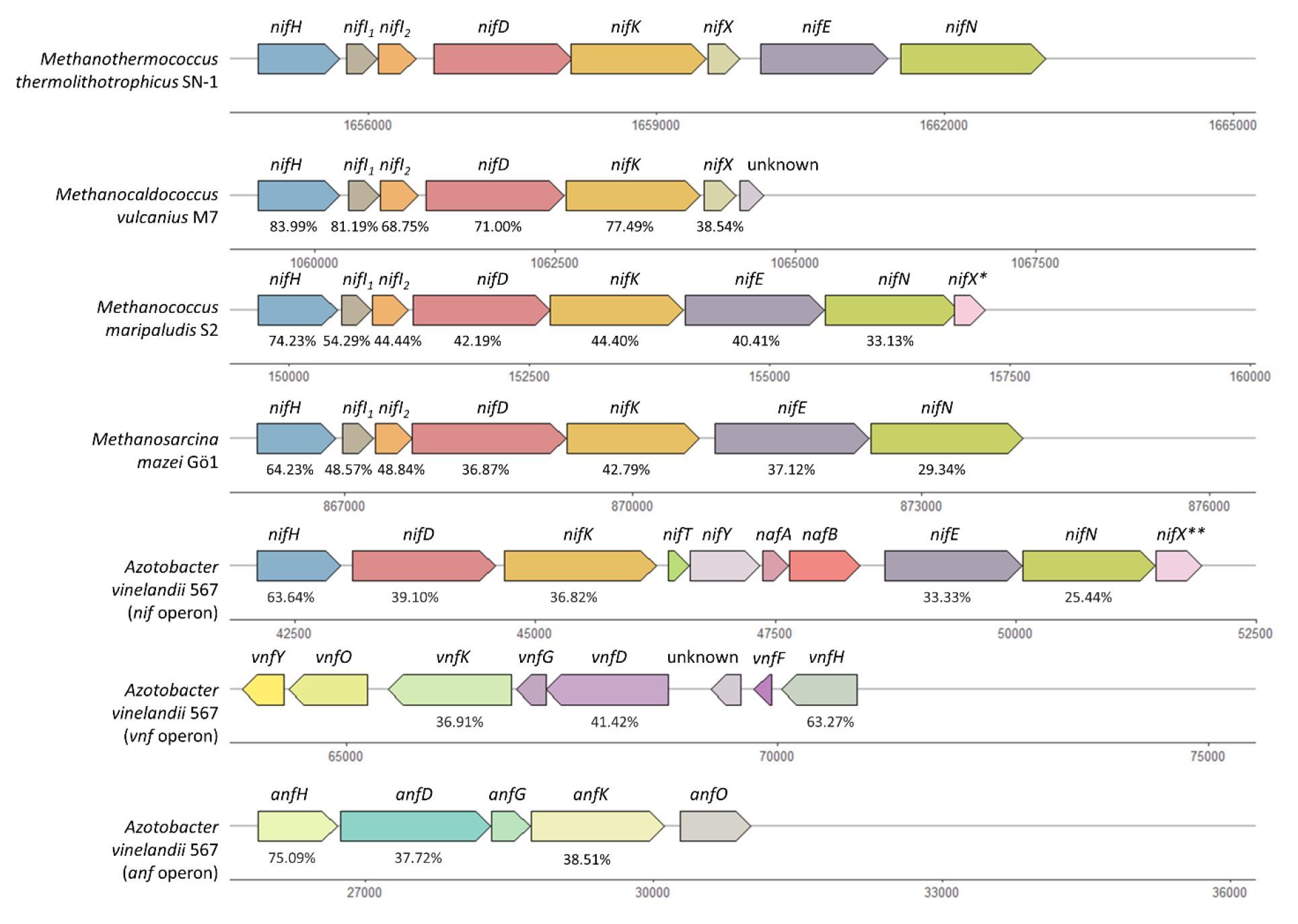

Supplement: FIG S1 [file mbio.02443-22-s0006.tif]

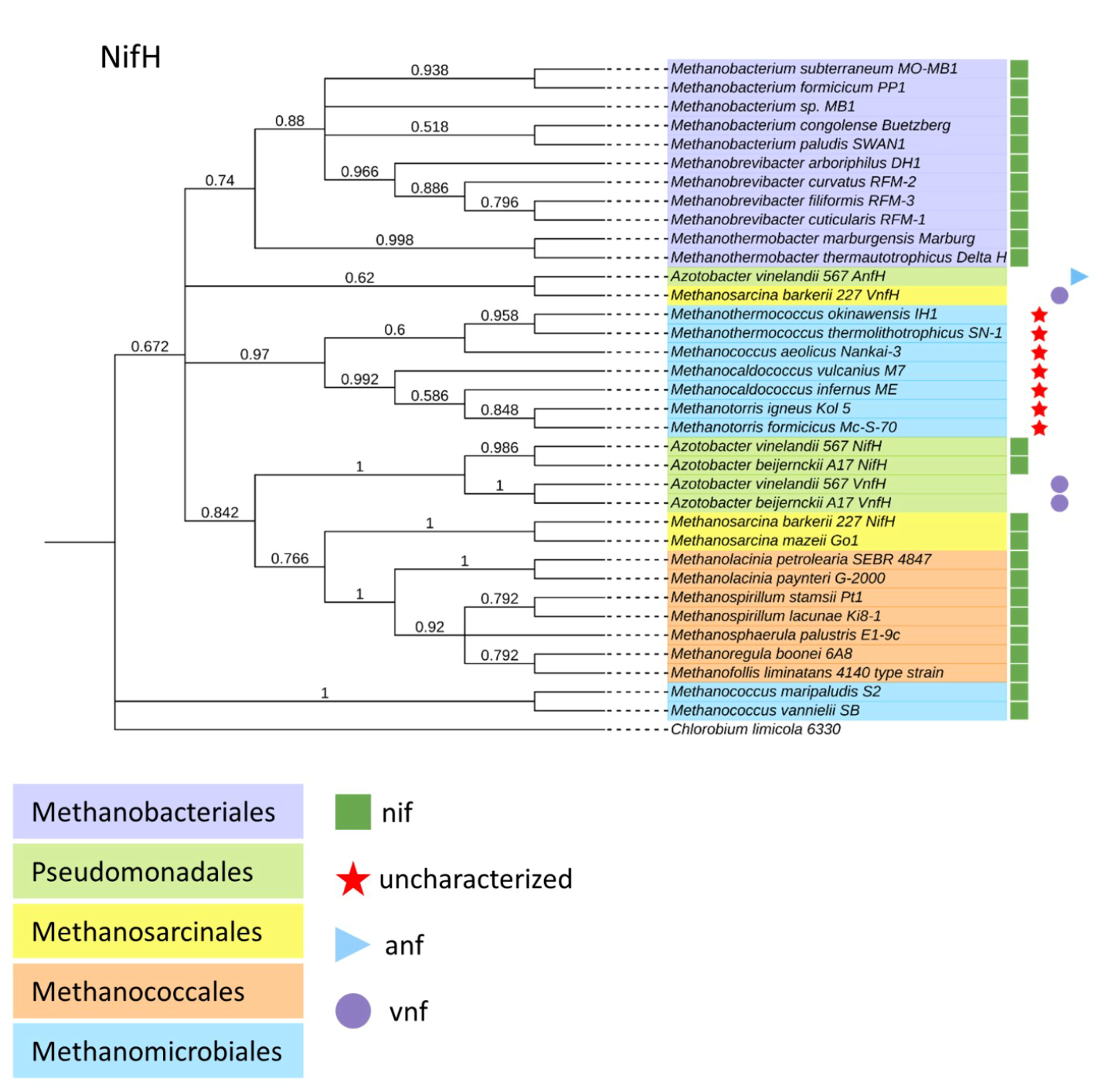

Supplement: FIG S2 [file mbio.02443-22-s0007.tif]

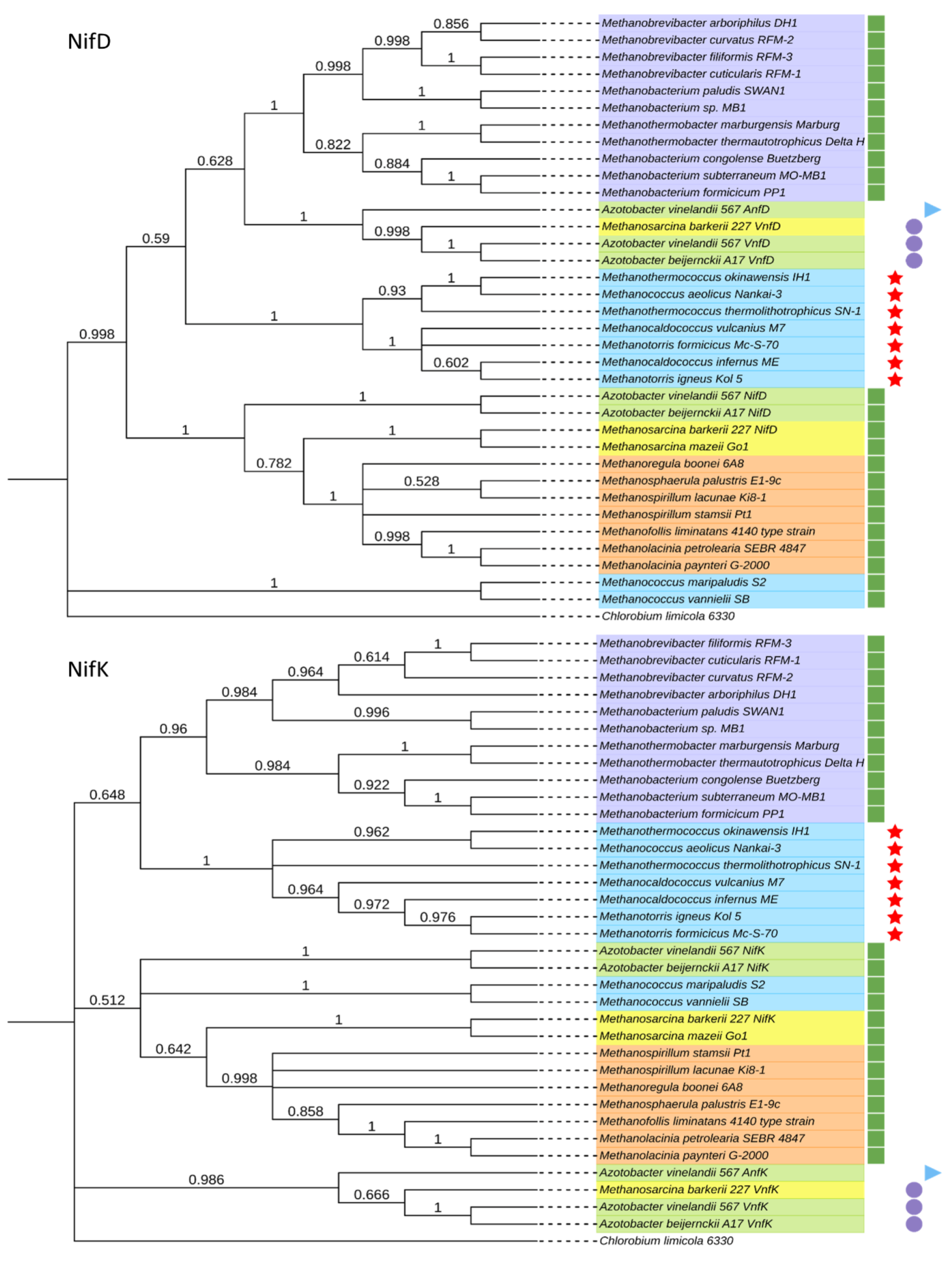

Supplement: FIG S3 [file mbio.02443-22-s0008.tif]

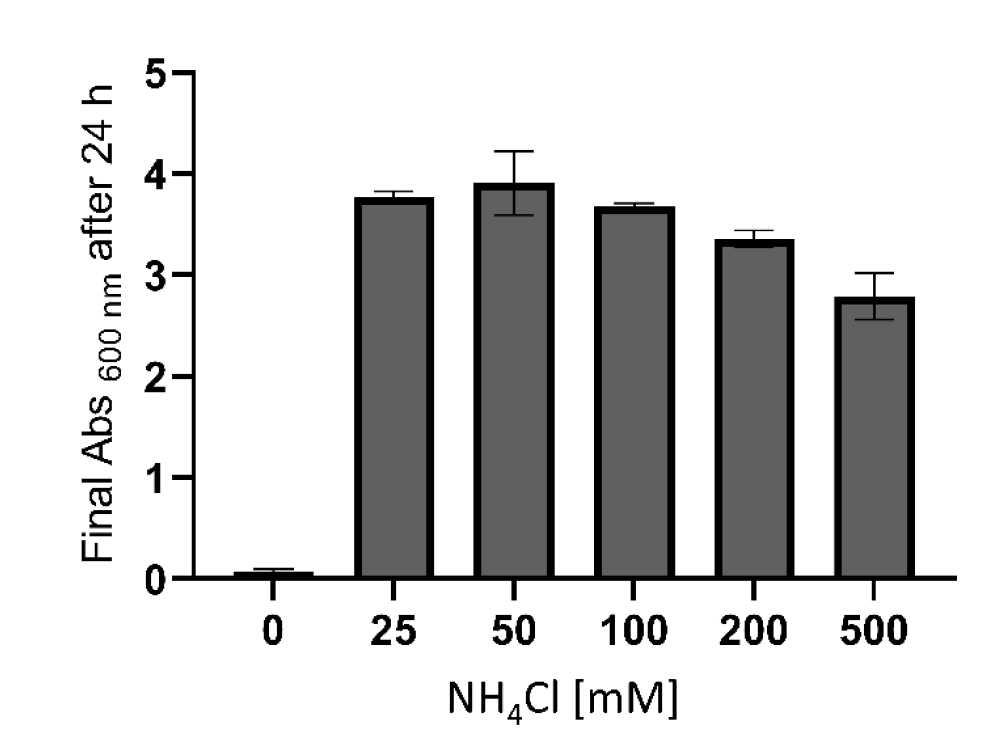

Supplement: FIG S4 [file mbio.02443-22-s0009.tif]

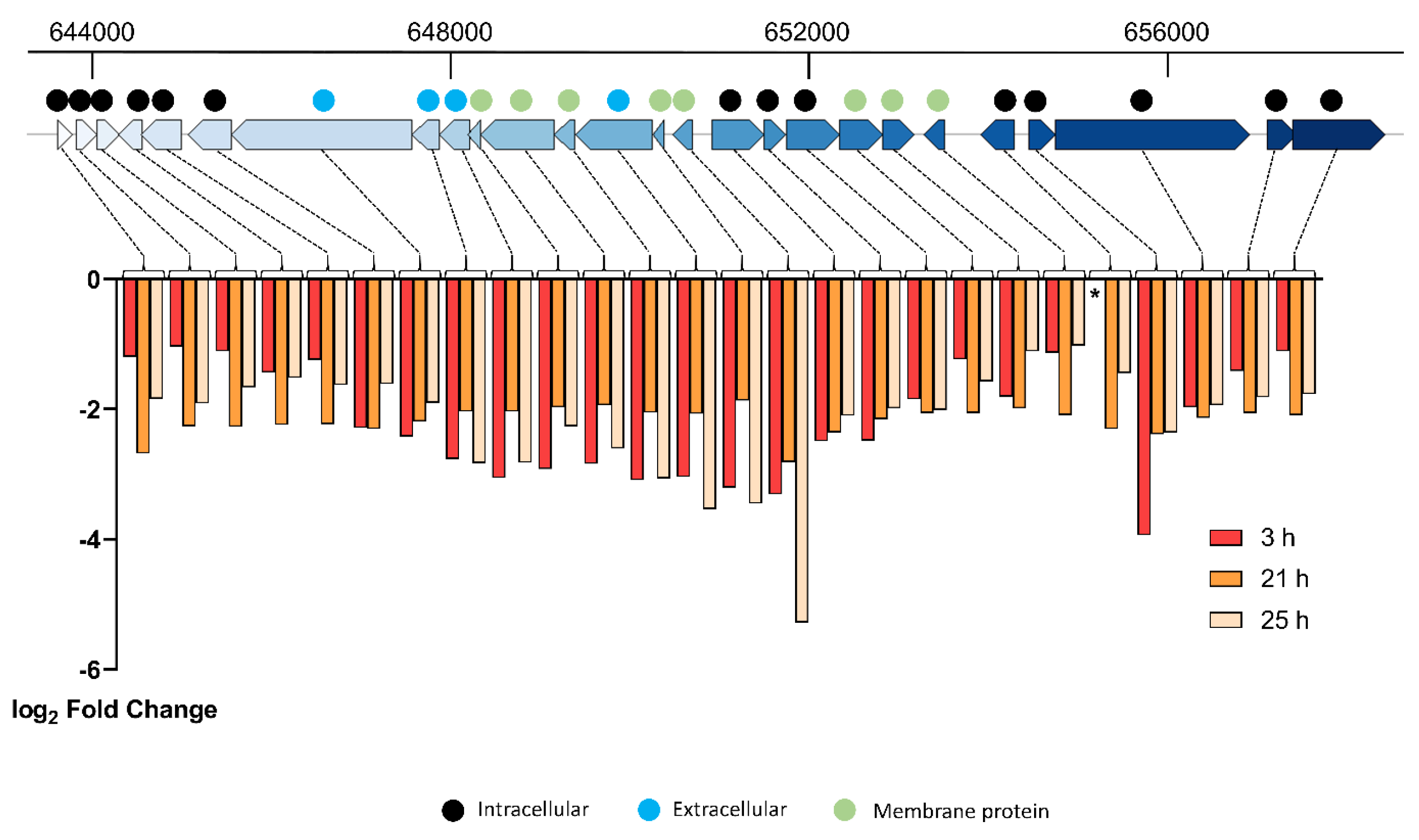

Supplement: FIG S5 [file mbio.02443-22-s0010.tif]
